# Response to 1-Year Fixed-Regimen Bevacizumab Therapy in Treatment-Naïve DME Patients: Assessment by OCT Angiography

**DOI:** 10.1155/2022/3547461

**Published:** 2022-02-21

**Authors:** Magdalena Hunt, Sławomir Teper, Adam Wylęgała, Edward Wylęgała

**Affiliations:** ^1^Chair and Department of Ophthalmology, Faculty of Medical Sciences in Zabrze, Medical University of Silesia, 40-760 Katowice, Poland; ^2^Department of Ophthalmology, District Railway Hospital in Katowice, 40-760 Katowice, Poland; ^3^Health Promotion and Obesity Management Unit, Department of Pathophysiology, Faculty of Medical Sciences in Katowice, Medical University of Silesia, Poland

## Abstract

**Purpose:**

To evaluate the effectiveness of intravitreal bevacizumab treatment in patients with diabetic macular edema (DME) by assessing retinal changes using optical coherence tomography angiography (OCT-A).

**Methods:**

This prospective study was performed in patients with treatment-naïve DME. The eyes of patients were imaged using a swept-source OCT system with a scan area of 6 × 6 mm. The DME patients with a central macular thickness (CMT) of ≥300 *μ*m received nine bevacizumab injections within 12 months. The demographic, systemic, and ocular parameters, including the best-corrected visual acuity (BCVA), CMT, microaneurysm (MA) count, and foveal avascular zone (FAZ) area in both superficial capillary plexus (SCP) and deep capillary plexus (DCP), as well as vessel density in SCP, were assessed in the patients. In addition, the response (good or poor) of the DME eyes to bevacizumab treatment and the final visual acuity (BCVA of 75 letters) were analyzed.

**Results:**

Seventy-seven eyes of DME patients were subjected to the final analysis. Bevacizumab treatment reduced CMT from 425.06 *μ*m (±77.15) to 350.25 *μ*m (±82.04) and improved BCVA by about 8.61 letters (from 64.73 to 73.34) in the patients. The mean number of MAs in SCP decreased from 3.51 ± 2.07 to 2.31 ± 1.15 (*p* < 0.001) and in DCP from 17.12 ± 11.56 to 12.21 ± 6.99 (*p* < 0.001), whereas the area of FAZ increased in SCP from 328.22 ± 131.38 to 399.70 ± 156.98 (*p* < 0.001) and in DCP from 571.13 ± 396.01 to 665.89 ± 412.77 (*p* = 0.001). The final BCVA letter score and CMT were statistically significant in both poor and good responders, as well as in BCVA < 75 and BCVA ≥ 75 groups.

**Conclusion:**

The fixed-regimen intravitreal bevacizumab therapy was effective in treating DME. Apart from noninvasive visualization of microvascular damage, OCT-A showed limited usefulness in predicting treatment response. Although the study showed that the number of MAs was significantly reduced during treatment, which is an OCT-A predictor of a good response to bevacizumab treatment at a 12-month visit, commonly observed artifacts may reduce the usefulness of OCT-A.

## 1. Introduction

Diabetic retinopathy (DR) is identified as one of the leading causes of preventable visual impairment and blindness worldwide [1–3]. It is estimated that the number of patients with diabetes mellitus will reach 429 million by 2030 [4] and increase further to 642 million by 2040 [5].

In the early stages of DR, microvascular damage, including the loss of pericytes and proliferation of endothelial cells, weakens the vascular walls, resulting in the formation of microaneurysms (MAs) and increasing the vascular permeability and pathologic neovascularization [6, 7]. The breakdown of the blood-retinal barrier caused by a high concentration of inflammatory mediators and the leakage of MAs lead to the development of diabetic macular edema (DME), which is identified as a leading cause of vision impairment in patients with diabetes mellitus type 2 [1, 8–11]. Because vascular endothelial growth factor (VEGF) is the dominant factor of retinal vascular hyperpermeability, DME is mainly treated using anti-VEGF inhibitors [3, 9, 12, 13]. Bevacizumab is a monoclonal, humanized VEGF-inhibiting antibody which is a common, off-label medication used for the treatment of DME [14–16] [17]. Although it is not approved for ophthalmology [16, 17], bevacizumab is often used as a first-line treatment in many countries. As the efficacy and safety of this drug have been less documented than approved drugs, even small-scale prospective studies may broaden the knowledge about optimal treatment regimens with bevacizumab for DME.

Currently, fluorescein angiography (FA) is the diagnostic gold standard for investigating the stages of DR [18]. By contrast, optical coherence tomography angiography (OCT-A) allows depth-resolved visualization of each retinal capillary layer without the need for dye injection [1, 12, 19–22]. The main advantage of this technique is that it enables three-dimensional imaging of retinal layers (superficial capillary plexus (SCP) and deep capillary plexus (DCP)) [3, 23].

In diabetic patients, OCT-A can reveal the enlargement of foveal avascular zone (FAZ), abnormalities in capillary flow density, and MAs, larger nonperfused areas, and neovascularization compared with controls. Studies have demonstrated more severe microvascular damage in DCP than in SCP in patients with DR [6, 8, 12, 24]. However, OCT-A artifacts are common and often observed as motion or doubling artifacts in the deeper layers, due to shadows in moving blood cells in the overlying retinal vessels [18, 24, 25].

This study is aimed at determining whether OCT-A can be a valuable tool for monitoring treatment with VEGF inhibitors in patients with DME. In addition, it also attempted to analyze whether predictive factors can be distinguished among the OCT-A parameters and characterize the dynamics of changes in the macular vascular network during bevacizumab treatment.

## 2. Materials and Methods

This prospective study was conducted among patients recruited from the ophthalmological outpatient clinic of the Clinical Department of Ophthalmology at the Faculty of Medical Sciences in the Medical University of Silesia, during 2018–2020. The study was conducted in accordance with the Declaration of Helsinki and approved by the Ethics Committee of the Medical University of Silesia (KNW/0022/KB1/126/I/18/19). All the included patients were clearly informed about the study, especially its purpose, protocol, and benefits as well as possible risks. Furthermore, written informed consent was obtained from all the participants.

The inclusion criteria for the study included the following: (1) patients with type 1 or type 2 diabetes mellitus, (2) age ≥ 18 years, (3) diagnosis of nonproliferative DR, (4) diagnosis of DME with a central macular thickness (CMT) of ≥300 *μ*m, (5) naïve to intravitreal treatment, and (6) best-corrected visual acuity (BCVA) of 24–78 ETDRS (Early Treatment Diabetic Retinopathy Study) letters. The exclusion criteria were as follows: (1) history of any retinal surgery; (2) previous intravitreal injections of anti-VEGF agents or steroids; (3) macular, focal, or pan-retinal laser photocoagulation; (4) eye conditions that interfere with imaging and affect visual acuity (e.g., cataract and cornea abnormalities); (5) diagnosis of glaucoma; (6) presence of epiretinal membrane, vitreoretinal traction in the macula, or other types of maculopathy unrelated to diabetes mellitus (e.g., age-related macular degeneration); (7) proliferative DR; and (8) unwilling to cooperate with OCT-A imaging.

All the participants were initially interviewed and then examined during the routine ophthalmologic visit. The following data were collected from them: age, sex, height, weight, concomitant medications and duration of diabetes mellitus, concomitant systemic diseases (e.g., hypertension, history of heart incidents and stroke, and chronic kidney disease), and serum level of glycated hemoglobin (HbA1c).

The participants were treated with intravitreal injections of bevacizumab. Anti-VEGF inhibitors were administered by an ophthalmologist in the ophthalmological outpatient clinic of the Clinical Department of Ophthalmology at the Faculty of Medical Sciences in the Medical University of Silesia. In each studied eye, nine injections of 1.25 mg/0.05 ml bevacizumab (Avastin) were administered over 12 months. The first five injections were given every month, and the subsequent four injections were administered every 2 months.

Before every injection, the patients were subjected to BCVA test, slit-lamp examination, and OCT and OCT-A analyses. The pupils of patients' eyes were dilated with 1% tropicamide before the OCT imaging. OCT-A was performed using a swept-source OCT (SS-OCT) system (DRI OCT Triton; Topcon, Inc., Tokyo, Japan) with a wavelength of 1050 nm at a speed of 100,000 A-scans per second (each 512 × 512 mm) [1, 3, 8]. Two fovea-centered OCT-A scans were taken with an area of 6 × 6 mm at baseline (up to 4 weeks before the first injection), during every visit up to 4 hours before intravitreal bevacizumab injection and up to 4 weeks after the last anti-VEGF injection. Only one OCT-A scan of better quality was assessed for every visit. The built-in IMAGEnet6 software (version 1.26.16898) was used for automated layer segmentation of retinal vasculature (SCP and DCP) [1, 3, 8]. The following boundaries were defined in segmentation: for SCP—2.6 mm below the internal limiting membrane to 15.6 mm below the junction between the inner plexiform and the inner nuclear layers; for DCP—15.6 mm below the inner plexiform and the inner nuclear layers to 70.2 mm below them [3, 23]. All the images were analyzed by two separate readers, and if the automated positioning or segmentation was recognized as inaccurate, manual corrections such as centration or propriete delineation of layers of the scans were made. The FAZ profile in the SCP and DPC was manually outlined using the freehand selection tool and was calculated by the build-in Topcon software [26]. Automated analysis by the build-in software was made for density, but microaneurysms were counted manually.

OCT-A scans within the area of 6 × 6 mm were assessed in quantitative and qualitative analyses. Quantitative analysis included the evaluation of vessel density in SCP, FAZ area, and the number of MAs in SCP and DCP [23]. The exclusion criteria for quantitative and qualitative analyses were as follows: (1) quality score of <40, (2) motion artifacts, (3) blurry images, (4) poor centration, (5) signal loss, and (6) images with segmentation error [1, 3]. Artifacts that prevented a reliable assessment were excluded.

In this study, DME was defined as a CMT of >300 *μ*m. Based on the response (poor and good) to anti-VEGF therapy, the study participants were divided into two groups. A good response to bevacizumab was defined as a reduction in the CMT of DME eyes by ≥10% after nine consecutive anti-VEGF injections compared to the initial value.

An additional analysis was also performed by dividing the patients into two groups: those who achieved a final visual acuity of ≥75 ETDRS letters and patients with a worse result.

### 2.1. Statistical Analysis

The changes in the OCT parameters were estimated using a mathematical equation derived for this purpose. Then, the relative FAZ area in SCP and DCP, relative MAs in SCP and DCP, relative CMT, relative BCVA (in EDTRS score), and all the analyzed densities were calculated according to the following equation to assess the effects of the therapy:

(1) $$\begin{array}{c} \text{Paramete}{\text{r}}_{\text{relative}}=\frac{{\text{Parameter}}_{\text{after therapy}}-{\text{Paremter}}_{\text{before therapy}}}{{\text{Parameter}}_{\text{before therapy}}}*100\%. \end{array}$$

Categorical variables were analyzed using the chi-squared test. The normality of the data was assessed using the Shapiro–Wilk test. Continuous variables measured during the 1-year period of the study were evaluated using repeated-measures analysis of variance (ANOVA). The association between continuous variables was investigated using Pearson's correlation or the Mann–Whitney test if applicable. Qualitative variables were assessed using the Spearman test followed by regression analysis. *p* values of <0.05 were considered significant. All analyses were performed using Statistica 13.3 (Tibco, Palo Alto, CA, USA).

## 3. Results

**Figure 1:**
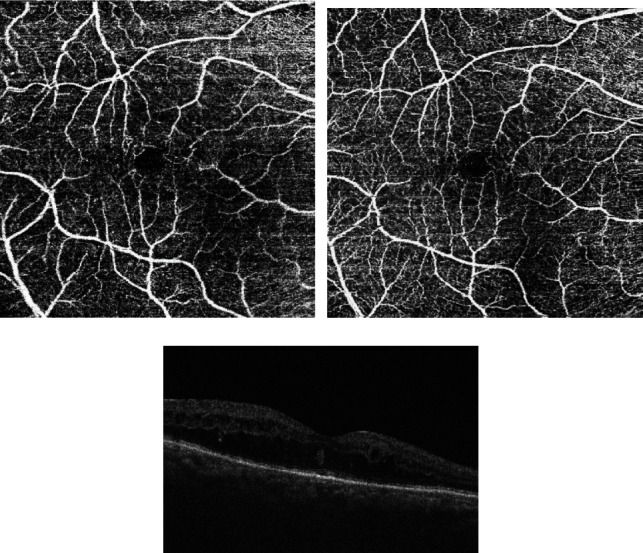
(a) Superficial capillary plexus of study patient before intravitreal treatment of bevacizumab. (b) Superficial capillary plexus of study patient after intravitreal treatment of bevacizumab. (c) Structural OCT B-scan before intravitreal treatment of bevacizumab.

**Figure 2:**
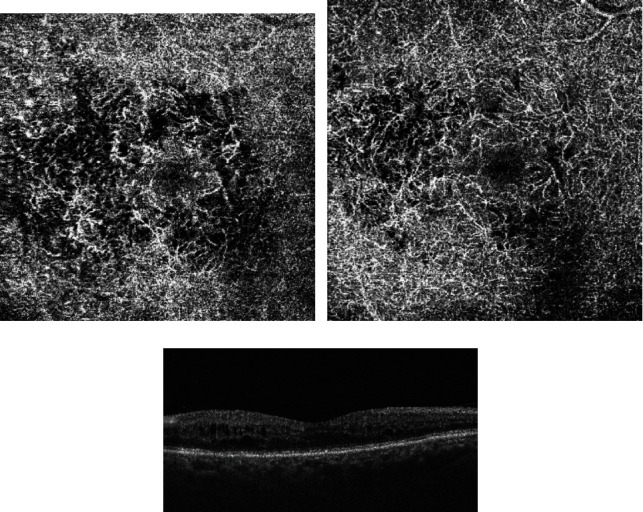
(a) Deep capillary plexus of study patient before intravitreal treatment of bevacizumab. (b) Deep capillary plexus of study patient after intravitreal treatment of bevacizumab. (c) Structural OCT B-scan after intravitreal treatment of bevacizumab.

A total of 116 eligible eyes of 112 patients with DME were analyzed in the baseline examination. We excluded 39 eyes (33.62%) as they were deemed unsuitable for OCT-A due to a low-quality score (14 eyes), blurry images (10 eyes), motion artifacts (7 eyes), poor centration (3 eyes), or signal loss (5 eyes). Finally, 77 eyes with DME were included in the final analysis (33 male (42.86%) and 44 female). The mean age of patients included in the study was 68.50 ± 8.53 years (47 to 84 years), and the mean HbA1c level was 7.06 ± 0.95% (5.50-9.40%). Patients had DM on average for 12.65 ± 7.74 (4.00-31.00 years). The mean BMI was 26.23 ± 3.98 (19.38-39.79). IOP was on average 15.74 ± 1.98 (12.00-21.00), while AL was 23.37 ± 0.68 mm(21.53-24.97) and the qualitative data are provided in Table 1. We performed our analysis in two stages. Firstly, we divided the study eyes based on the response to bevacizumab: poor response—25 eyes and good response—52 eyes. Secondly, we divided all the study participants based on the final BCVA of 75 ETDRS letters. Forty eyes achieved a final BCVA of ≥75 letters in the ETDRS chart, while 37 eyes showed a BCVA of <75 letters. If both eyes were eligible for the study, we selected the one with more severe retinal edema. In 8% of patients, the OCT-A images were manually corrected with inaccurate automated positioning and segmentation of OCT-A scans. OCT-A images of the superficial capillary plexus (SCP) and deep capillary plexus (DCP) with structural OCT B-scans before and after the intravitreal treatment of bevacizumab are presented in Figures 1(a)–1(c) and 2(a)–2(c).

The data collected from medical history showed no significant difference between the patients with a BCVA of <75 letters and those with a BCVA of ≥75 letters in terms of general medical and ophthalmological variables. Similarly, no difference was observed between good and poor responders (Table 1). In addition, patients from the BCVA ≥ 75 group were significantly older 70.8 ± 9.59 vs. 66.03 ± 6.47 years (*p* = 0.019), and their diabetes was significantly shorter 10.85 ± 6.44 vs. 14.59 ± 8.61 years (*p* = 0.034). Furthermore, both baseline CMT 365.78 ± 70.86 vs. 463.51 ± 62.33 *μ*m (*p* < 0.001) and ETDRS 69.85 ± 5.75 vs. 59.19 ± 4.83 (*p* < 0.001) and final CMT 280.5 ± 47.7 vs. 384.03 ± 66.88 *μ*m (*p* < 0.001) and final ETDRS score 67.08 ± 4.04 (*p* < 0.001) values differed significantly between the BCVA > 75 and the BCVA < 75, respectively. The only significant differences between the good and poor responders were found in the final values of CMT 317.83 ± 55.76 vs. 417.68 ± 55.76 (*p* < 0.001) and ETDRS 73.71 ± 7.98 vs. 72.56 ± 7.98 (*p* < 0.02).

The OCT-A parameters were compared between the DME eyes that responded well and the DME eyes that responded poorly to anti-VEGF inhibitors. In both groups, the mean area of FAZ and the mean number of MAs were observed to be higher in DCP compared to SCP. Furthermore, independent of the response to bevacizumab treatment, the mean number of MAs was decreased in SCP from 3.51 ± 2.07 to 2.31 ± 1.15 (*p* < 0.001) and in DCP from 17.12 ± 11.56 to 12.21 ± 6.99 (*p* < 0.001) in both groups. The FAZ area increased in SCP from 328.22 ± 131.38 to 399.70 ± 156.98 (*p* < 0.001) and in DCP from 571.13 ± 396.01 to 665.89 ± 412.77 (*p* = 0.001).

A significant reduction in the number of MAs in SCP and DCP was observed after fixed-regimen bevacizumab therapy, which confirmed that DR did not progress in the treated DME patients (with the simultaneous absence of other markers of progression such as hemorrhages and IRMA not found in the fundus images).

### 3.1. Repeated-Measures ANOVA in All Participants

**Figure 3:**
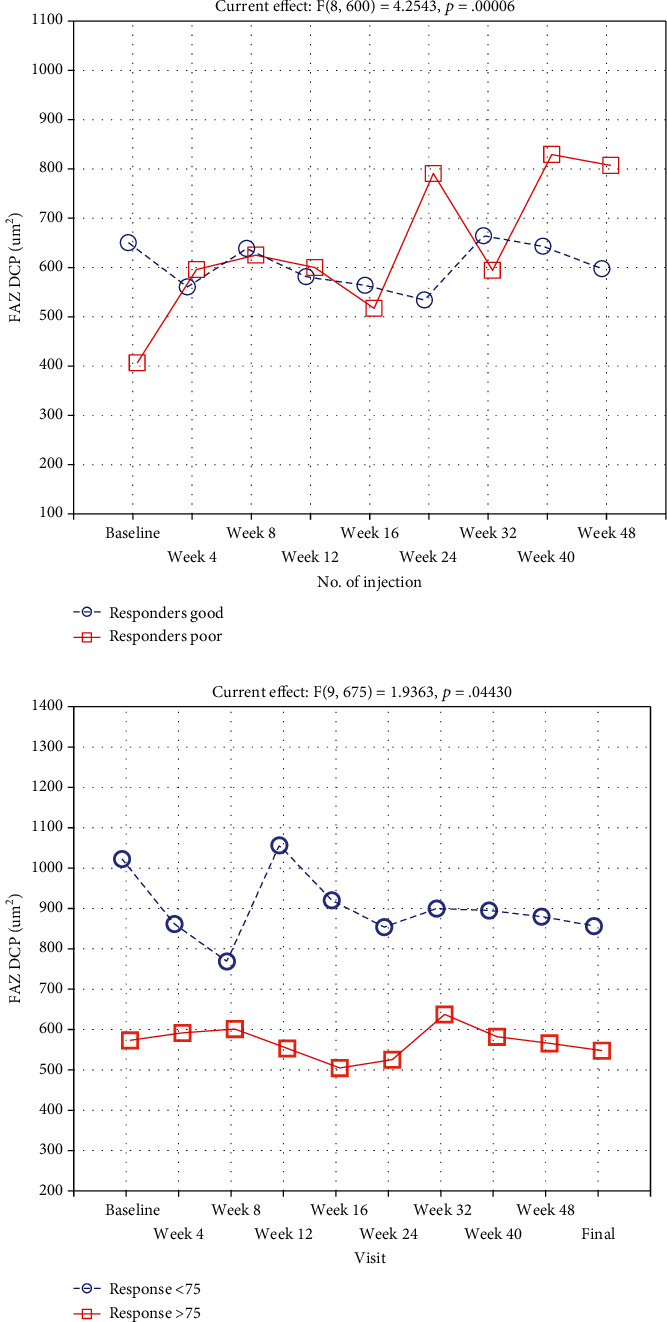
Size of FAZ in DCP in the BCVA < 75 (*N* = 37) and BCVA ≥ 75 (*N* = 40) groups and in the DME patients described as poor (CMT reduction < 10%, *N* = 52) or good (CMT reduction > 10%, *N* = 25) responders during subsequent intravitreal bevacizumab injections.

**Figure 4:**
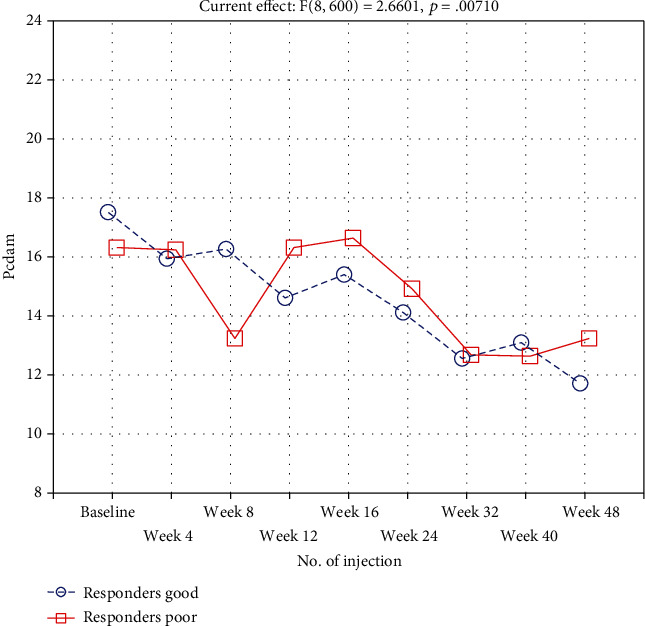
Number of MAs in the DCP group and in the DME patients described as poor (CMT reduction < 10%, *N* = 52) or good (CMT reduction > 10%, *N* = 25) responders during subsequent intravitreal bevacizumab injections.

**Figure 5:**
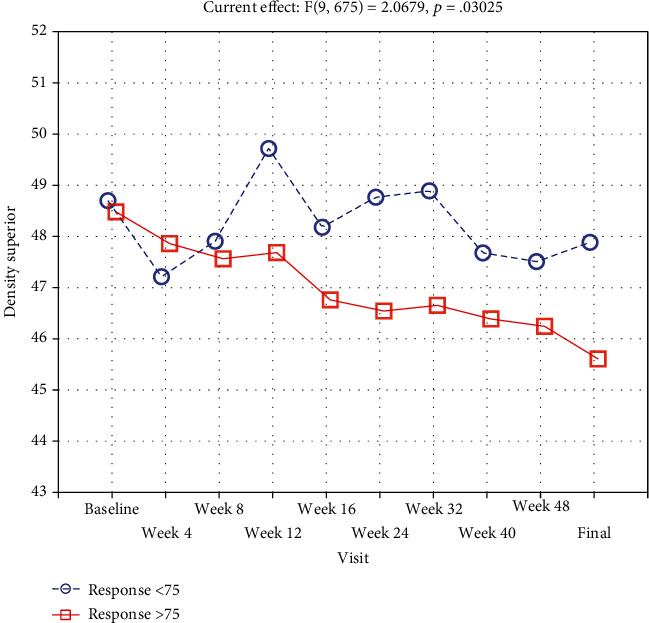
Density in the superior, quadrant of DME patients during subsequent intravitreal bevacizumab injections.

Repeated-measures ANOVA revealed that a range of ocular biometric parameters (BCVA: *p* < 0.001, FAZ area in SCP: *p* < 0.001, and FAZ area in DCP: *p* < 0.001) showed significant changes over 1 year of the study (Figures 1 and 2). Moreover, vascular density average was found to be decreased significantly during the treatment (*p* < 0.001; nasal density: *p* = 0.003, superior density: *p* < 0.001, and temporal density: *p* < 0.001) (Figure 3). However, neither total nor inferior density exhibited any significant interaction (*p* = 0.79 and *p* = 0.84, respectively). In addition, repeated-measures ANOVA revealed that the injections significantly reduced the number of MAs in SCP and DCP (Figure 4). Similarly, a significant reduction was noted in CMT and ETDRS in both groups after intravitreal anti-VEGF treatment (Figure 1). The changes observed in FAZ areas are displayed in Figure 3 and density in Figure 5. The variations found in MAs and FAZ area in the SCP and DCP, as well as in nasal, temporal, and superior vessel density in the SCP, indicated significant changes in the macular vascular network during the treatment.

### 3.2. Repeated-Measures ANOVA in All Participants

To measure the changes during treatment between two groups, a repeated measurement ANOVA was conducted. Repeated-measures ANOVA revealed that a range of ocular biometric parameters (BCVA: *p* < 0.001, FAZ area in SCP: *p* < 0.001, and FAZ area in DCP: *p* < 0.001) showed a significant increase over 1 year of the study (Figure 3). Moreover, vascular density average was found to be decreased significantly during the treatment (*p* < 0.001; nasal density: *p* = 0.003, superior density: *p* < 0.001, and temporal density: *p* < 0.001).

However, neither total nor inferior density exhibited any significant interaction (*p* = 0.79 and *p* = 0.84, respectively). In addition, repeated-measures ANOVA revealed that the injections significantly reduced the number of MAs in SCP and DCP (Figure 4). Similarly, a significant reduction was noted in CMT and ETDRS in both groups after intravitreal anti-VEGF treatment. The decrease found in MAs and FAZ area in the SCP and DCP, as well as in nasal, temporal, and superior vessel density in the SCP, indicated significant changes in the macular vascular network during the treatment.

### 3.3. Repeated-Measures ANOVA between the Groups

A significant increase in the poor responders compared to a decrease in good responders was noted in the FAZ area in DCP (*p* < 0.001), while the FAZ area in SCP did not differ between the groups (*p* = 0.51). Both FAZ areas in DCP and SCP were measured consecutively during the study that decreased significantly between BCVA < 75 and BCVA ≥ 75 groups (*p* = 0.007 and *p* = 0.044 for SCP and DCP, respectively) (Figure 3).

Furthermore, vascular density superior was found to significantly decrease weaker in the BCVA < 75 group than in the BCVA ≥ 75 group (*p* = 0.03) (Figure 5). All other vascular densities did not differ significantly.

On the other hand, an opposite trend was noticed between the good and poor responders, where changes in nasal density were not found to be significant.

### 3.4. Correlations

We also investigated the correlations in the course of bevacizumab treatment and at the end of the study.

The analysis of variables measured in all the included patients showed that relative BCVA strongly negatively correlated with CMT (*r* = –0.41, *p* < 0.001) in good responders, while in poor responders, the correlation was very weak (*r* = –0.01, *p* < 0.001). On the other hand, a positive correlation was found between the FAZ area and MAs measured in SCP (*r* = 0.44).

A positive linear correlation between relative BCVA (a percentage change between the EDTRS score at the last visit and at baseline) and relative FAZ area (a percentage change between the size of FAZ at the final visit and at baseline) in DCP was indicated. Our analysis revealed that for each 20% increase in the relative FAZ area, a 0.8% decrease in relative BCVA could be expected. In addition, we found that HbA1c% negatively correlated with oral treatment (*r* = –0.41) and relative BCVA (*r* = –0.23), while positively correlated with hypertension (*r* = 0.48) and relative density superior (*r* = 0.28). Relative CMT correlated with other parameters, and the number of MAs in DCP was associated with the baseline ETDRS score (*r* = –0.30, *p* = 0.018) and the MA count in the posterior zone (average correlation, *r* = –0.377, *p* < 0.001). No other correlations were found between the diabetes parameters and OCT-A findings, except for the correlation between HBA1c% and relative density superior and relative density inferior (*r* = 0.24, *p* = 0.034 and *r* = 0.28, *p* = 0.016, respectively). We also found no relationships between oral treatment, gender, insulin treatment, combined treatment, hypertension, ischemic heart disease, brain stroke or lens status, and the OCT-A parameters, apart from the associations between insulin treatment and relative MAs in DCP (*r* = 0.23, *p* < 0.05) and lens status (*r* = –0.25) and between relative MAs in SCP, lens status, and superior density (*r* = 0.33). Relative MA count in DCP was strongly correlated with relative FAZ area in DCP in the BCVA < 75 group (*r* = 0.46), whereas in the BCVA ≥ 75 group, the correlation was weak (*r* = –0.07). Furthermore, the correlation between the relative MAs in SCP and the relative FAZ area in SCP was strong in poor responders in comparison to good responders.

A regression analysis was performed to identify the factors affecting the changes in MA distribution in DCP.

In the BCVA ≥ 75 group, for every 1% increase in relative FAZ area in SCP, a 0.23% increase in DCP MAs was expected, while a 1% increase in total relative density was predicted to lead to a 0.30% decrease in DCP MAs. In the case of the BCVA < 75 group, for every 0.58% increase in relative MAs in DCP, a 1% increase in relative CMT was expected, while a 1.53% decrease was predicted to lead to a 1% increase in relative density in the inferior quadrant (Table 2).

## 4. Discussion

Our study aimed to demonstrate the effectiveness of 12-month intravitreal bevacizumab therapy in patients with DME and their response to the treatment. The effectiveness of the anti-VEGF treatment was evaluated morphologically by performing OCT-A and SS-OCT. The results showed that intravitreal bevacizumab therapy effectively reduced DME. A significant improvement in BCVA and reduction in CMT were found after fixed-protocol intravitreal bevacizumab injections. In addition, no progression of DR was noticed in patients with DME after the treatment. The mean number of MAs in both SCP and DCP was also decreased in patients regardless of their classification.

Our study showed that the fixed protocol of treatment with 9 bevacizumab injections within 12 months was helpful in the improvement of visual acuity and in reduction of central macular thickness in the study group of patients with diagnosed DME. Other studies have reported that intravitreal anti-VEGF injections can reduce microvascular damage caused by diabetes mellitus. The Diabetic Retinopathy Clinical Research Network qualified 660 patients with DME and randomly assigned them to treatment with anti-VEGF agents such as aflibercept, bevacizumab, or ranibizumab. At baseline, the mean visual acuity letter score of the patients was determined at 64.8 ± 11.3, and the mean central subfield retinal thickness at 412 ± 130 *μ*m. The bevacizumab group received 10 injections on average within 1 year of the study with improvement of 9.7 in the BCVA letter score, and the mean CMT reduction was 101 ± 121 *μ*m [14]. These findings do not differ significantly from that of our study, especially the improvement in the visual acuity letter score. However, the studies differ in the type of anti-VEGF treatment applied and the number of injections administered and it is difficult to compare the two studies. On the one hand, Sarda et al. reported that the mean visual gain observed in their study was +10.1 ETDRS letters and reduction in CMT was 65.1 *μ*m after 5 aflibercept or ranibizumab intravitreal injections [27]. On the other hand, Călugăru et al. demonstrated that the mean BCVA improved and CMT decreased significantly compared to the baseline values after anti-VEGF therapy in anatomic nonresponders and responders [28]. As in other studies, Vujosevic et al. observed the significant improvement in BCVA with mean change of 13 ± 10 ETDRS letters after treatment. In that study, naïve patients were treated either single DEX-I 0.7 mg (Ozurdex, Alergan, Inc., Irvine, California, USA) or 3 monthly IVR 0.5 mg (Lucentis, Novartis, Genetech, San Francisco, USA) [26].

MAs are visible lesions often observed in the early stages of DR that could be visualized using OCT-A. Moreover, these lesions can be located precisely within the retinal vasculature by OCT-A [18, 29]. Our results showed that, regardless of division into groups, in all patients, the mean number of MAs was higher in DCP than in SCP, and MA count was decreased after intravitreal bevacizumab treatment in comparison to baseline. Moreover, we noted that poor-responding DME eyes had a higher number of MAs in DCP than good responders after treatment. Similar observations were reported by Lee et al. who observed that the number of MAs was increased to a great extent in DCP in comparison to SCP after three consecutive injections of different kinds of anti-VEGF agents. Division into poor and good responders was based on the reduction in CMT by >50 *μ*m after the treatment and also compared their results with a group of control eyes. However, no significant differences were noticed between good and poor responders in the SCP parameters [12, 18]. In our study, we found that the mean number of MAs decreased in both SCP and DCP, apart from the response to bevacizumab treatment. Hasegawa et al. observed 77.3 ± 8.1% of MAs in DCP in DME eyes, whereas in DME eyes with a CMT of >400 *μ*m, 91.3 ± 9.1% of MAs were located in DCP [13].

Pongsachareonnont et al. demonstrated that the number of MAs was reduced in SCP and DCP (40% relative to baseline) after a single anti-VEGF injection of aflibercept, bevacizumab, or ranibizumab and ranibizumab was the most effective among the three studied drugs. Anti-VEGF agents helped decrease the MA count in association with a reduction of CMT and improvement of BCVA [8]. Ho et al. observed that MAs were more clearly delineated in the 6 × 6 mm scans compared with 3 × 3 mm scans. Discrepancies can be found among studies regarding the detectability of MAs between FA and OCT-A images, and it was pointed out that not all MAs visualized on FA images were identified on OCT-A images [18]. Salz et al. showed that SS-OCT-A had a sensitivity of 85% and a specificity of 75% compared to FA [18, 30].

Furthermore, Falavarjani et al. reported no significant difference in the FAZ area and vessel density in patients with macular edema after a single intravitreal anti-VEGF injection. However, they indicated that more injections should be conducted to confirm their results [18, 31]. In our study, we found that the FAZ area was larger in DCP than in SCP, apart from the response to the treatment, which is also in line with the results of Lee et al. Moreover, poor responders showed a larger FAZ area in DCP compared to good responders. After bevacizumab treatment, an increase in the FAZ area was noted in both SCP and DCP compared to baseline, which contradicts the results of Pongsachareonnont et al., who reported a significant reduction in the FAZ area in both plexuses after injection of anti-VEGF agents [8].

Another parameter assessed in OCT-A images is the vascular perfusion density at the macula in eyes with DR. Vessel density is the proportion of blood vessel area and total scanned area [29]. Our study showed that total vessel density in SCP decreased after bevacizumab treatment. According to Sorour et al., the values of macular vessel density in SCP, DCP, and total capillary plexus did not significantly differ between baseline and after one, two, or three injections of anti-VEGF agents in both 3 × 3 and 6 × 6 mm scans [9].

OCT-A can quickly illustrate the microvascular abnormalities in both SCP and DCP in diabetic patients in a noninvasive way. Unfortunately, artifacts are a major limitation of the OCT-A examination. Our study noted motion artifacts, blurry images, low-quality scores, and images with a segmentation error. Other researchers have shown that motion and doubling artifacts were relatively high in both 3 × 3 and 6 × 6 mm scans [25].

We also analyzed the ocular, systemic, and demographic parameters of participants in our study. The patients in the BCVA ≥ 75 group were significantly older, and their diabetes was significantly shorter. No other correlations were found between the diabetes parameters and the OCT-A findings except that between HBA1c% and density superior and density inferior, which may be a random observation, considering the number of analyzed parameters. In addition, no relationship between gender, type of treatment, systemic factors, or lens status and the OCT-A parameters was found, apart from the associations between insulin treatment and relative MAs in DCP and lens status, and between relative MAs in SCP, lens status, and superior density. Tang et al. analyzed 434 OCT-A images of SCP obtained in patients with different stages of DR to assess the biomarkers of DR. They found that OCT-A metrics were related to the severity of DR but not to the presence of DME. Increased FAZ area was associated with a shorter axial length and decreased CMT. Nevertheless, OCT-A parameters were not correlated with age, duration of diabetes, and systemic factors (e.g., blood pressure, lipids, estimated glomerular filtration rate, and body mass index) [1].

We conducted a 12-month prospective study on DME patients who were treated with fixed-regimen intravitreal bevacizumab injections. Only treatment-naïve patients were included, and all of them received nine bevacizumab injections, while in many other studies, patients received fewer injections of anti-VEGF inhibitors [12, 18, 27, 28]. In addition, both the number of injections and follow-up period and the number of assessed parameters were lower in other studies compared to our study. Moreover, we obtained OCT-A images in every visit of patients. Usually, OCT-A scans with an area of 3 × 3 mm are used for analysis, while we used 6 × 6 mm scans in our study. However, a relatively high percentage of inaccurate images was obtained which did not allow us to perform reliable examinations as the scans had projection and motion artifacts and images obtained from the DME patients were of lower quality.

## 5. Study Limitations

The study has several noticeable limitations. First, the number of participants was significantly reduced due to the low quality of the obtained OCT-A images. Second, we measured OCT-A parameters in SCP and DCP only in 6 × 6 mm scan frame and not in other frames. Third, we only included the eyes for which good-quality images and good fixation were achieved, and thus, the generalizability of our findings was limited [3]. Fourth, quantitative evaluation of the MA count and FAZ area was a subjective analysis because no reliable software is currently available for this purpose [12].

## 6. Conclusions

This study analyzed whether fixed-protocol intravitreal bevacizumab therapy is effective in treating DME and whether OCT-A is a useful tool to observe the changes in the clinical features of DR. Although OCT-A allows noninvasive monitoring of changes in the vascular network of the macula, it has limited usefulness in predicting the response to treatment. A more effective reduction in the MA count during treatment is an OCT-A predictor of a good response of DME eyes to bevacizumab. However, artifacts are very common in the OCT-A images of DME patients and can significantly reduce the usefulness of this modality. The factors that contribute to achieving a satisfactory visual acuity (>75 ETDRS letters) are higher baseline BCVA and less severe macular edema, as well as older age and longer duration of the disease. These could indicate that DME is less aggressive in form, given that only previously untreated patients are included in the study.

## Figures and Tables

**Table 1:** Qualitative data of patients described as poor (CMT reduction < 10%, *N* = 52) and good (CMT reduction > 10%, *N* = 25) responders and in the BCVA < 75 (*N* = 37) and BCVA ≥ 75 (*N* = 40) groups receiving intravitreal bevacizumab treatment. The data were compared using the chi-squared (*χ*^2^) test. Statistical significance was set at *p* < 0.05.

	Response < 75	Response > 75	Row	*χ* ^2^	*p*	Responders good	Responders poor	Row	*χ* ^2^	*p*
Per os treatment										
Absent	13	19	32	1.21	*p* = 0.27134	21	11	32	0.09	*p* = 0.76308
Present	24	21	45			31	14	45		
Totals	37	40	77			52	25	77		

Insulin										
Absent	30	31	61	0.15	*p* = 0.69879	41	20	61	0.01	*p* = 0.90698
Present	7	9	16			11	5	16		
Totals	37	40	77			52	25	77		

Gender										
Male	15	18	33	0.16	*p* = 0.69279	21	12	33	0.40	*p* = 0.52719
Female	22	22	44			31	13	44		
Totals	37	40	77			52	25	77		

Chronic kidney disease										
Absent	34	36	70	0.08	*p* = 0.77295	46	24	70	1.16	*p* = 0.28127
Present	3	4	7			6	1	7		
Totals	37	40	77			52	25	77		

Hypertension										
Absent	13	16	29	0.19	*p* = 0.65981	20	9	29	0.04	*p* = 0.83465
Present	24	24	48			32	16	48		
Totals	37	40	77			52	25	48		

Ischemic heart disease										
Absent	28	34	62	1.07	*p* = 0.30199	45	17	62	3.70	*p* = 0.05444
Present	9	6	15			7	8	15		
Totals	37	40	77			52	25	77		

Myocardial infarction										
Absent	33	35	68	0.05	*p* = 0.81770	48	20	68	2.48	*p* = 0.11548
Present	4	5	9			4	5	9		
Totals	37	40	77			52	25	77		

Brain stroke										
Absent	34	39	73	1.23	*p* = 0.26790	50	23	73	0.59	*p* = 0.44184
Present	3	1	4			2	2	4		
Totals	37	40	77			52	25	77		

Lens status (phakic/pseudophakia)										
Phakic	24	25	49	0.05	*p* = 0.82936	31	18	49	1.12	*p* = 0.28474
Pseudophakia	13	15	28			21	7	28		
Totals	37	40	77			52	25	77		

Laterality										
OD	19	23	42	0.29	*p* = 0.58825	25	17	42	2.70	*p* = 0.10017
OS	18	17	35			27	8	35		
Totals	37	40	77			52	25	77		

Combined treatment										
Absent	31	30	61	0.90	*p* = 0.34255	42	19	61	0.23	*p* = 0.62910
Present	6	10	16			10	6	16		
	37	40	77			52	25	77		

**(a):** 

	Response ≤ 75 *R* = 0.77058035; *R*^2^ = 0.59379408; adjusted *R*^2^ = 0.45839211 *F*(9, 27) = 4.3854; *p* < 0.00132; Std. error of estimate: 23.721					
	*b* ^∗^	Std.Err.	*b*	Std.Err.	*t* (27)	*p* value
Intercept			-5.02	10.76	-0.47	0.64
CMT relative	0.13	0.29	0.29	0.66	0.44	0.67
ETDRS %	-0.21	0.23	-0.94	1.04	-0.90	0.37
FAZ SCP %	0.34	0.15	0.23	0.11	2.20	0.04
FAZ DCP %	0.12	0.19	0.06	0.10	0.61	0.54
Density %	-0.32	0.15	-0.30	0.14	-2.09	0.05
Density sup %	0.17	0.19	0.79	0.87	0.90	0.37
Density inf %	-0.45	0.21	-1.31	0.59	-2.21	0.04
Density nasal%	0.18	0.16	1.03	0.91	1.13	0.27
Density temporal%	0.13	0.18	0.59	0.87	0.68	0.50

**(b):** 

	Response ≥ 75 *R* = 0.61835710; *R*^2^ = 0.38236551; adjusted *R*^2^ = 0.19707516 *F*(9, 30) = 2.0636; *p* < 0.06629; Std. error of estimate: 29.124					
	*b* ^∗^	Std.Err.	*b*	Std.Err.	*t* (30)	*p* value
Intercept			1.24	10.34	0.12	0.91
CMT relative	0.58	0.24	1.03	0.43	2.37	0.02
ETDRS %	0.11	0.23	0.36	0.77	0.47	0.64
FAZ SCP %	-0.14	0.19	-0.10	0.14	-0.72	0.48
FAZ DCP %	-0.36	0.24	-0.20	0.13	-1.50	0.14
Density %	-0.12	0.18	-0.41	0.63	-0.65	0.52
Density sup %	0.26	0.19	0.90	0.65	1.39	0.17
Density inf %	-0.45	0.20	-1.53	0.67	-2.28	0.03
Density nasal%	0.17	0.28	1.05	1.69	0.62	0.54
Density temporal%	-0.27	0.25	-1.78	1.65	-1.08	0.29

## Data Availability

Data are available upon reasonable request.
